# Eat like a Pig to Combat Obesity

**DOI:** 10.3390/metabo13030420

**Published:** 2023-03-13

**Authors:** Theo A. T. G. van Kempen, Ruurd T. Zijlstra

**Affiliations:** 1 Department of Animal Science, North Carolina State University, Raleigh, NC 27695-7621, USA; 2 Department of Agricultural, Food and Nutritional Science, University of Alberta, Edmonton, AB T6G 2P5, Canada

**Keywords:** obesity, glycemia, epigenetics, humans, swine

## Abstract

Obesity and related metabolic health issues are a growing human threat, with many theories regarding its causes. In swine, physiologically alike to humans, considerable knowledge on obesity mechanisms has been accumulated. Calorie counting is the basis for managing swine diets and applied with great accuracy. Epigenetic programing predisposes pigs to insulin insensitivity, but pigs seem to sense this insensitivity and consequently eat less, preventing obesity. Pigs naturally prefer to eat small breakfasts and large dinners. Deviating from this eating pattern or providing diets with a high glycemic burden can trigger obesity; however, pigs will restrict food intake to prevent serious obesity. Interestingly, in practice, problems with obesity are rarely seen, even when pigs are fed poorly timed diets similar to junk food, likely because swine diets are balanced for every nutrient. Indeed, feeding pigs diets deficient in micronutrients does trigger obesity. For humans, several micronutrient requirements have not been set officially, and diets optimized for all micronutrients are rarely provided. In conclusion, various obesity triggers are being debated for humans, which have been proven in swine. Obesity problems in pigs are nevertheless less excessive, likely because pigs recognize unhealthy eating practices and consequently reduce food intake to avoid serious complications. Finally, swine diets are normally balanced for all nutrients, which may be an important practice to prevent obesity, from which human health could greatly benefit.

## 1. Introduction

Over the decades, numerous diets aimed at combatting obesity have risen and fallen in popularity. The concept of ‘eating like a pig’, however, has not been considered, to our knowledge. Pigs are typically portrayed as dirty, sloppy and obese; and if people call others ‘pigs’, it generally has a strongly negative connotation. Interestingly, pigs are inherently rather clean animals. Winston Churchill had an interesting quote: ‘I like pigs: cats look down on human beings, dogs look up to them, but pigs just treat us as their equals’. Physiologically, Churchill was exactly right.

Nutritionally, pigs and humans have a lot in common. In the wild, both would have consumed omnivore diets, scavenging a variety of plant products ranging from roots to fruit, as well as eggs, insects, honey and the like, and eating meat whenever it was accessible [1]. Their digestive tracts, consequently, are remarkably similar [2,3], to the point that swine are high on the list of candidates as cross-species organ donors for man [4]. Digestibility studies confirm this similarity: the digestibility coefficients match reasonably well [5]. Last but not least, metabolically, again, there is great similarity. For example, prior to the introduction of biotechnology-produced insulin, pig-derived insulin was the primary treatment for type I diabetes, and digestive enzymes harvested from porcine pancreases are used to treat human pancreatic insufficiency.

A marked difference between modern man and pig is that modern man typically eats a diet driven by emotions and at times of the day that ‘fit’ with societal norms intertwined with convenience. Palatability trials with pigs indicate that they, e.g., dislike certain flavors; but when there is no choice, they eat a diet that is not their first choice. Moreover, pigs try hard to consume food at times that match their physiological needs.

Modern pigs are raised for meat production. In those production systems, food makes up roughly 70% of the total production costs. Consequently, a tremendous effort is put into understanding the fundamentals of nutrition and how this can be translated into diets that are most efficiently converted into muscle tissue. Adipose tissue also has value, as it harbors a lot of the meat’s taste, but given society’s drive for low-fat foods, swine production has adapted to producing pork with a low-fat content.

This knowledge has been converted into databases and computer models, so that diets can be optimized for maximum efficiency (e.g., INRAPorc from van Milgen and Noblet, [6]; Watson from Ferguson [7]). These models are continuously refined using data from large numbers of pigs, which followed exactly the prescribed diets. In strong contrast, controlled human studies are generally conducted with very small populations of subjects for very short durations, as emotions come into play. Humans are hard to persuade to follow a boring research diet, especially over a longer period of time, and humans are easily tempted to deviate from a protocol if peer pressure or desires are high. Even worse, in survey type experiments, people are asked what they ate for the past one or several days, and these data may well be subsequently extrapolated to patterns of behavior and then correlated with developments in health. Recalling what and especially how much a person ate yesterday is already a challenge for many, confounded even further by the desire not to include items that may be considered less acceptable. Extrapolating these data to lifetime impacts is, in our eyes, rather risky.

In man, obesity and related type II diabetes is the health challenge for the 21st century [8]. A tremendous effort is put into understanding its causes and into finding solutions that ideally fit within the emotional constraints of a person. A number of theories have been proposed explaining obesity. What we will try to do next is to review several of those theories from the perspective of nutritionists whose work has focused on swine.

## 2. Calorie Counting

The energy content of foods is measured in calories (or joules) and can be determined accurately using a so-called bomb calorimeter. In such an instrument, a food item is simply burned, and the released energy (heat) is measured. This energy value represents the amount of gross energy a person would consume when eating such a food. Foods are typically not 100% digestible, while digestion itself also requires energy, which is ultimately dissipated as heat. Finally, there are urinary and gaseous losses of energy, albeit normally small.

In human nutrition, most calculations appear to be performed with the total energy contents of foods (e.g., EFSA and USDA dietary guidelines). Although efforts have been made to determine digestible energy content both in vivo (in actual people) and in vitro (in a test tube), these efforts have not been translated into comprehensive datasets, which allow a nutritionist to calculate the energy content of complex diets. Actually, most datasets on the nutritional value of foods aimed at humans are incomplete to the point that they provide little robust value for performing diet optimizations (e.g., USDA, NEVO). An important reason for this is that man has access to an enormous number of ever-changing foods, making it practically impossible to characterize them all.

**Figure 1:**
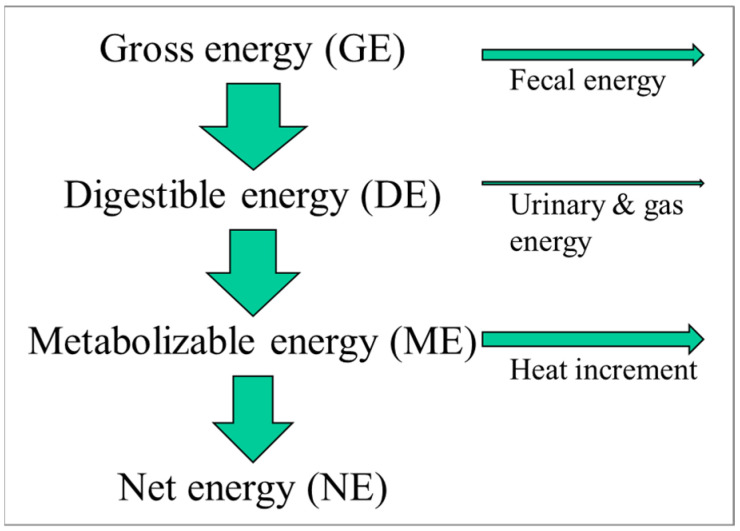
Energy utilization in foodstuffs.

For pigs, in contrast, only a limited number of dietary ingredients are used. For example, in the USA, pigs are typically fed a diet consisting of maize, soybean meal, and in recent decades, a maize-derived coproduct of ethanol production called dried distillers’ grains with solubles. The limited number of ingredients that change at a relatively slow pace with time has allowed the characterization of each of those ingredients in terms of its energy utilization by the animal (Figure 1). We thus differentiate between the gross energy content of the food (as determined by a bomb calorimeter), the digestible energy content (subtracting fecal energy), the metabolizable energy content (subtracting the urinary and gaseous (e.g., methane) energy losses), and lastly, the net energy content. The net energy content is the amount of energy the pig has available to support metabolism or growth, e.g., to exercise or grow tissue, such as muscle or adipose tissue. As an example, maize contains the following in kcal/kg: gross energy, 3933; digestible energy, 3451; metabolizable energy, 3395; and net energy, 2672 [9]. Not only have the energy inputs into the pigs been carefully characterized but also the energy outputs. The latter can be performed with a tool called an indirect calorimeter. Based on experiments with those indirect calorimeters, we know the energetic cost of exercise, of growing muscle or adipose tissue, of lactating, etc.

**Figure 2:**
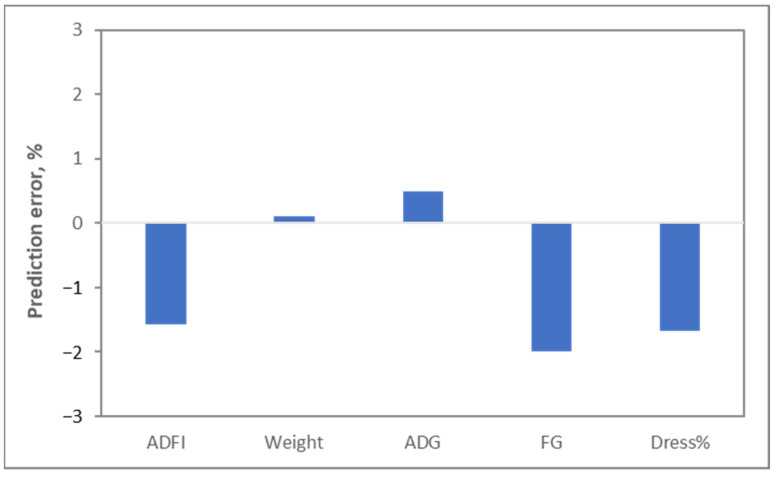
Prediction error between modeled estimates of pig food intake (ADFI: average daily food intake), gain (ADG: average daily gain), weight, efficiency of conversion of food into weight gain (FG: ADFI/ADG ratio) and dressing percentage (Dress%: yield of carcass as percentage of body weight). Ferguson, personal communication.

As indicated above, the data from all these sources have been included in computer models, which are used to determine what pigs all around the world should be fed, whether they are in Europe, where wheat is the primary cereal, or in the US, where maize is the primary cereal, or whether pigs are genetic lines selected for reproduction, meat production or not genetically improved at all, or whether pigs are infant, adult, male or female. Even more importantly, these models do a fantastic job in predicting what is happening with energy. A nice example is the Watson model developed by Ferguson [7]. Validations of this model indicate that food intake can be predicted with an error of less than 2%, and weight gain can be predicted with an error of less than 0.5% (Figure 2).

This returns us to the discussion on calories in versus calories out. Given the accuracy of these models, the answer to this discussion is quite easy. Energy metabolism is a reasonably simple calculation exercise in which the energy consumed, in the form of net energy, needs to find an outlet. These outlets can be tweaked, e.g., by exercising. The energy that is ‘left over’ will be used for growth. When the animal is a human being past puberty, most likely, this growth will be in the form of (typically undesirable) adipose mass. One must take into account the fact that a good basis of calorie counting exists, and arguably, a lot of obesity problems can be averted by either reducing calorie intake and/or by increasing calorie output, such that a net negative energy intake can be created.

These models, in practice, are not perfectly black and white. After all, there are some nuances in energy metabolism, which these models do not track. For example, a more recent finding is that the conversion of dietary net energy into ATP, the most important energy carrier within biology, is not as fixed as once believed [10]. The change is associated with the health of mitochondria; when mitochondria function sub-optimally, this conversion can be as much as 25% less efficient than what many textbooks claim. Humans affected by this would generate more heat, and consequently, at the same energy intake, they have less energy available for becoming obese. The opposite, though, is difficult to find. It takes energy to deposit adipose tissue, and this energy can come from only one source: what we consume.

Another factor to consider is that for pigs, we are typically interested in a relatively short period of time, i.e., months. In humans, we hope for a healthy lifespan of close to a century. An error in the models of 1 g of adipose tissue per day would mean about 150 g of fat on a 100 kg pig; this is practically negligible. An error of 1 g for a human over a period of 60 years (post-puberty) equates instead to 22 kg of fat, enough to make one obese.

Nevertheless, we would argue that swine nutritionists in general believe that calorie counting would also apply to humans. The parameters in the equations would certainly be affected by sex, age and genotype. This may not be 100% accurate, and there may be a rare exception, but for the public at large, eating less and/or exercising more would achieve a negative energy balance and consequently a loss of adipose tissue weight.

## 3. Genetics

In humans, genetic variation exists, and this can certainly affect energy metabolism. In pigs, the same is the case. Two extreme examples are Piétrain and Meishan pigs. The former are typically very muscular (as if they worked out in the gym on a daily basis); the latter are typically chunky little pigs (as if they were couch potatoes). These distinct genetic lines differ in how the energy is utilized in the body. In the very lean Piétrain pigs, heat losses to the environment are higher under cold conditions, as they have limited insulation reserves that adipose tissue would provide. When energy is available for tissue growth prior to reaching sexual maturity, this energy is directed predominantly to the growth of muscles. In the Meishan pigs, the ability to grow muscle tissue is much more limited, and they mature faster. Consequently, in practice, a Meishan pig quite often appears to be obese.

The above rules of energy metabolism, nevertheless, still apply to both genetic lines of pigs. There are small differences in the model factors used. For example, van Milgen and Noblet (Table 1), in developing the INRAPorc model, determined that in (castrated) Meishan pigs, the maintenance requirement was 799 kJ/kg^0.643^, while this value was 762 for the Piétrain pigs (intact males), a difference of 5% (the maintenance requirement is the energy needed to not change weight, assuming limited physical activity and assuming that no food is being digested). Van Milgen and Noblet also have factors (c and d) describing how growth changes with age, and for that, there are big differences between the two lines. The efficiency of energy utilization for protein and fat deposition, however, was not sufficiently different between the two lines to assign separate factors. This would indeed mean that genetics does affect energy utilization and, in particular, muscle growth potential. The basic energy rules, though, vary very little.

Notably, the k_f_ is close to 1 (0.916), while the k_p_ is only 0.511. What these values mean is that for every 1 kcal of net energy consumed, 0.916 kcal of fat can be formed; in contrast, if that 1 kcal was used for protein synthesis, then only 0.511 kcal of protein could be formed. The conversion of dietary energy into fat is thus extremely efficient. Fat has a high energy content, approximately 9 kcal/g; adipose tissue can actually replace water, so a person gaining 1 kg due to fat may have gained 10,000 kcal. Protein, in contrast, has an energy value upon catabolism of only 4 kcal (assuming nitrogen is converted into urea). Protein gain is invariably associated with water gain. Muscle typically consists of roughly 25% protein and 75% water. This means that a person gaining 1 kg of muscle has gained only 1000 kcal of usable energy. Protein tissue is also energetically expensive to maintain, as there is a continuous turnover of proteins, which requires energy. Adipose tissue, in contrast, has little turnover and consequently requires very little energy to maintain.

These strong differences in energy content per kg of body weight do mean that an obese person who starts exercising and dieting can actually gain body weight if adipose tissue is broken down while muscle tissue is accreted. The high energy content of adipose tissue also means that, in extreme obesity cases, a person can carry a year’s worth of energy in the form of adipose tissue, illustrating how hard it can be to correct serious obesity.

## 4. Sex

Just as genetics have an effect on energy metabolism, so does sex. In Table 1, for the Large White genetic line, the coefficients are included for males, females and castrated males. Just like for genotypes, the factor a, the maintenance requirement, varies due to sex. Male Large White pigs have the lowest maintenance requirement, while castrated males have the highest maintenance requirement; this actually implies that the males can eat the least if they want to maintain a steady weight. The three sexes do vary in how much protein is accreted during development (factors c and d). However, the efficiency of energy use for protein (k_p_) and fat (k_f_) deposition is, again, not different.

## 5. Epigenetic Programing

In recent decades, we have learned that the expression of genes, as inherited from our parents, can be tweaked without altering the genes themselves. This is called epigenetic programing. There are a couple of time points during our development when epigenetic programing is very important. This includes certain phases of fetal development and around birth. Adverse conditions in those periods can trigger this epigenetic programing, which, among others, can make people more prone to type II diabetes [11]. A textbook example of epigenetic programing is the Dutch hunger winter of 1944–1945, toward the end of World War II. People born following this hunger winter were observed to be more prone to type II diabetes and obesity because their mothers experienced famine during a specific period of their fetal development. This triggered epigenetic programing, which affected insulin sensitivity for life [12].

**Figure 3:**
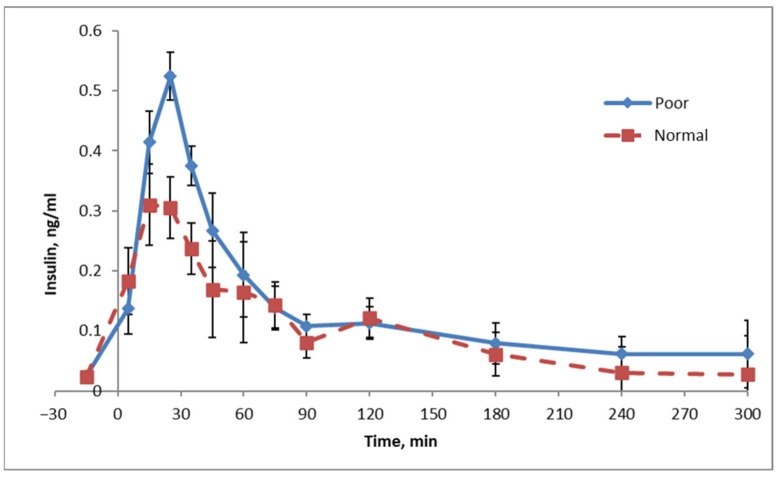
Insulin response to a glycemic challenge in piglets with a normal birth process and growth (‘Normal’) or with a birth process hampered by dystocia and, subsequently, poor growth (‘Poor’) [13].

Epigenetic programing also occurs in pigs, both during fetal development and during the birth process. Inadequate nutrition and hypoxia (a lack of oxygen) appear to be important triggers. The pigs that suffer from this programing grow slower, and they tend to have higher body fat contents. To characterize this, we followed the birth process of a large group of piglets to identify both piglets with a normal birth process and those with a challenging birth process, which caused hypoxia. We subsequently tracked the growth of these pigs, and, as expected, the piglets that had a difficult birth process grew notably slower than piglets with a normal birth process [13]. At the age of 10 weeks, we subsequently submitted these piglets to a glycemic challenge. In both groups of pigs, the blood glucose response to this challenge was the same. However, the pigs with the difficult birth process mounted a significantly stronger insulin response to this glycemic challenge (Figure 3). This implies that, as is the case with type II diabetes, these piglets had compromised insulin sensitivity [13]; the pigs had to produce significantly more insulin to trigger the metabolic processes required to process the incoming glucose. Children with growth retardation show similar changes in insulin and glucose [14].

**Figure 4:**
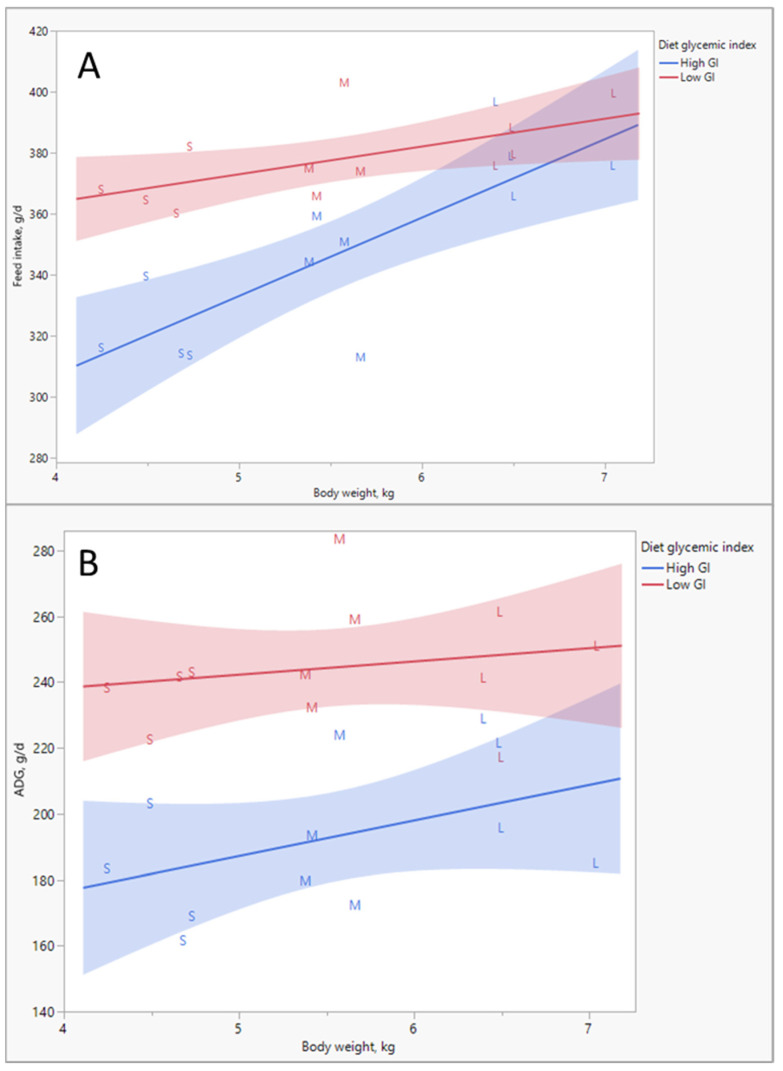
(**A**) Food intake versus body weight (BW) of Small (S), Medium (M) and Large (L) piglets at weaning when fed a diet with a low or high glycemic index (GI). (**B**) Gain (ADG, g/d) in piglets selected at weaning as small or as big as affected by the glycemic index of the diet. Small pigs represent a high proportion of animals predisposed to type II diabetes due to epigenetic programing [15].

In strong contrast to human beings, though, we seldom find pigs that become severely obese as a consequence of adverse fetal or birth conditions. The reason for this appears to be that the pigs with compromised metabolic health seem to restrict their food intake, presumably to prevent excesses in blood glucose. Interestingly, when these pigs are placed on diets with a lower glycemic load, they actually increase their intake (Figure 4A) [15]. More importantly, on the low glycemic diet, they also grew nearly as fast as their healthy siblings (Figure 4B). This implies that the glycemic load of the normal diet was a burden to these diabetes-prone piglets, which was sensed by the animals themselves; taking away this burden by lowering the glycemic load of the diet allowed the piglets to eat and develop normally!

What these observations mean is that some humans may well be able to blame their early life for their obesity problems: epigenetic programing occurred and predisposed them to obesity and type II diabetes. Pigs under those conditions, though, seem aware enough of this problem, such that they reduce their food intake to prevent them from becoming obese, especially when this food has a high glycemic index, which would trigger adiposity if consumed at high rates. Foods with a low glycemic load, however, did not require such downshifts in food intake to prevent hyperglycemia and allowed piglets to grow normally. In modern man, we seem to have lost this ability to sense whether the diet induces unhealthy hyperglycemia!

## 6. Glycemic Response

**Figure 5:**
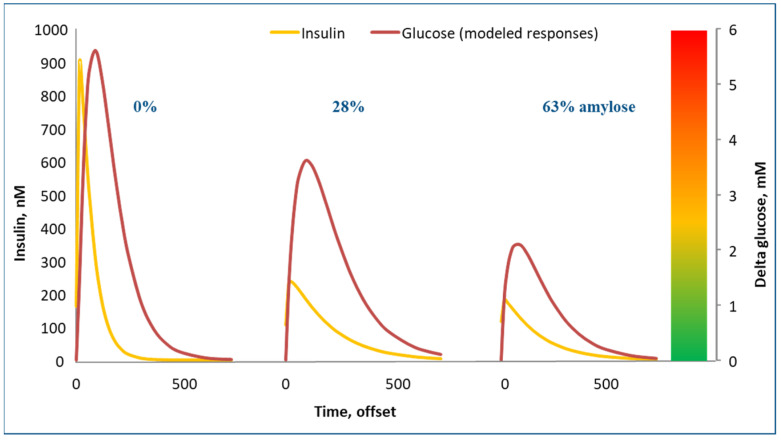
Glucose (mM above baseline) and insulin (nM) response to standardized meals in pigs fed diets varying in amylose content of starch (diets themselves were 70% starch) [16,17]. The colored bar is a subjective attempt to indicate how safe the various increases in levels of blood glucose over baseline are.

The glycemic response is the rise and fall in blood glucose observed after consuming a (standardized) meal containing a source of glucose (Figure 5). Effectively, the glycemic response is a function of the amount of glucose in a diet, e.g., as starch or sucrose, and how easily/quickly these components are broken down into glucose in the intestinal tract and subsequently absorbed (the latter is very fast).

The glycemic response of a food is interesting, as our bodies try to prevent strong rises in glucose. High levels of glucose are not healthy, as glucose can react with other molecules in the body, resulting in unhealthy products, such as advanced glycation end (AGE) products [18]. The lead mechanism for controlling blood glucose is through secretion of the hormone insulin. Insulin effectively tells the body to start metabolizing glucose. This can be for producing heat to stay warm, but it can also be for the production of fat. The latter, generalizing, occurs when the body is overloaded with glucose. Technically, if we were able to prevent blood glucose from rising above this threshold, we would prevent the production of excess fat.

**Figure 6:**
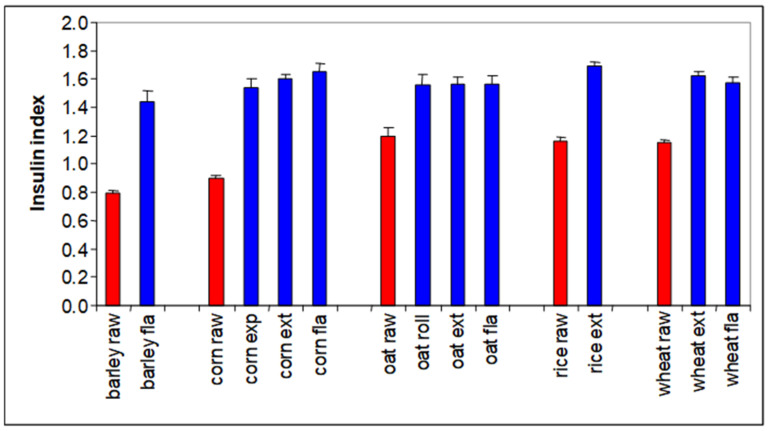
Glycemic response to various cereal and cereal products, as used in swine nutrition (insulin index is the maximal rate of in vitro glucose release/min, which corresponded with the in vivo insulin response) [15,16].

The glycemic response of a diet is thus an important parameter in our fight against obesity. As explained, it is composed of two factors: how much glucose is consumed as, e.g., digestible starch or sugar, and how quickly this glucose is released into the blood stream. The glycemic response to a diet has received a lot of attention in both human and pig nutrition. For pigs, a database was constructed with over 700 samples of common dietary ingredients for which we had characterized this glycemic response (Figure 6) [15]. We performed this using a lab assay developed for use in human nutrition but which we also validated in actual pigs [16,17]. The pig data indeed indicated that some starches break down extremely fast after eating, giving a strong glycemic response, while others are broken down very slowly and also very poorly, and they thus hardly provide a glycemic response. Figure 5 shows the blood glucose and insulin response to three starch sources. On the left in the graph, waxy maize starch (0% amylose) yielded very strong glucose and insulin peaks. On the right, high amylose (63%) maize starch resulted in very modest glucose and insulin peaks. Technically, it would be very interesting to switch our convenience food industry away from low amylose starches to high amylose starches, as they yield much lower glycemic responses and as they induce physical satiety for a much longer period of time. The crops to support this are already available, but they are not grown on a large scale. Swine trials with high amylose starches indeed confirmed these benefits [15].

Notably, slowly digestible for starches also implies poorly digestible. For high amylose maize starch, a substantial portion of the starch is not digested but fermented by microbes in the large intestine [17]. Fermented means that the starch is broken down by microbes, especially in the large intestine. Upon this breakdown, volatile fatty acids are formed but also heat. Thus, the net energy of the diet, as explained above, becomes lower. These slowly digestible starches are thus interesting in theory for preventing obesity. We indeed completed several piglet experiments, which confirmed this effect: exchanging rapidly digestible starches for slowly digestible starches resulted in leaner pigs!

**Figure 7:**
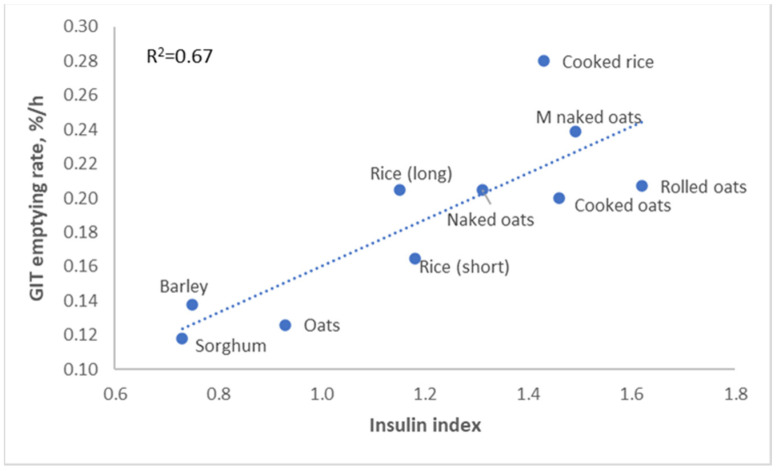
Relation between insulin index and gastro-intestinal tract (GIT) passage rate of cereal grains [19].

Rapidly digestible starches can trigger adiposity through their high glycemic index, but they also result in very rapid passage through the small intestine (Figure 7). This means that they induce physical satiety only for a short period of time; our stomachs will start growling for food much quicker after a meal based on rapidly digestible starches.

**Figure 8:**
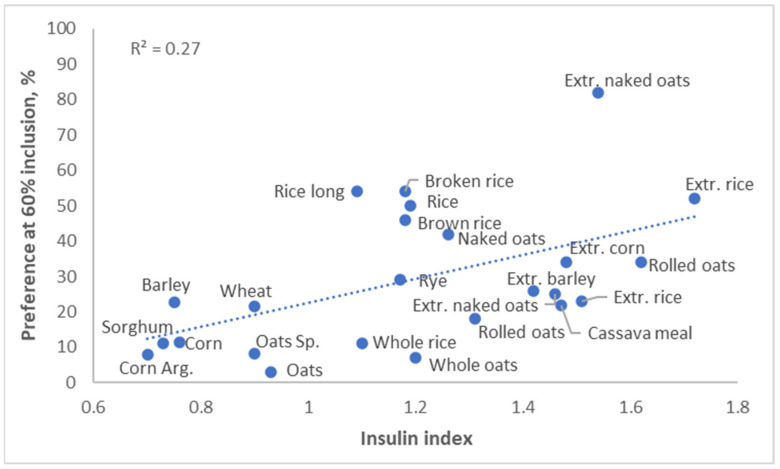
Palatability versus insulin index of a variety of foodstuffs (Solà-Oriol and van Kempen, unpublished).

The glycemic response to a diet affects not only obesity, but it also affects palatability. Pigs preferred diets with a high insulin index, and humans appear to have the same preference (Figure 8, Solà-Oriol and van Kempen, unpublished). This is quite logical; a high glycemic index generally results in a food tasting sweet, and both pigs and humans have a serious sweet tooth.

High glycemic foodstuffs thus have three downsides against them:

- High palatability, which stimulates intake;
- Rapid intestinal passage rate, which induces limited physical satiety;
- Strong stimulation of insulin and blood glucose, which triggers conversion of glucose into fat.

Sweeteners are a way of tricking us into thinking that a product has a high glycemic index but without providing a strong glycemic response. Technically, this sounds like an interesting solution. Physiologically, however, a big question is how our bodies respond to those sweeteners. Our work implied that the insulin response in pigs is already steered by the diet’s glycemic response, as measured by taste receptors in the mouth (Figure 5) [16]. This remains a point of debate, but if the sweet taste does indeed induce insulin, but subsequently, our bodies do not receive the glucose to be processed under the influence of this insulin, is that a sign to the body to ignore the insulin signal?

**Figure 9:**
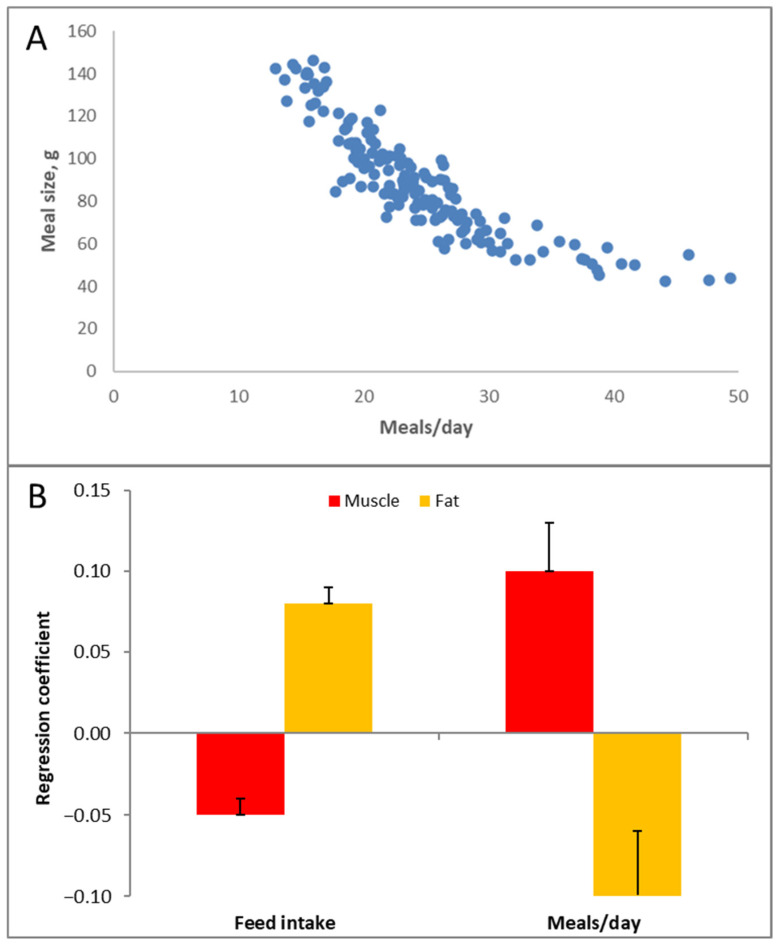
(**A**) Variation in the number of meals per day and meal size (multiplication of which yields food intake) observed in a trial with grow–finish pigs (each dot is a pig) and (**B**) their relationship with muscularity and adiposity (fat) at slaughter.

The glycemic response is not only a function of the type and amount of carbohydrates in the diet but also of meal size. The larger the meal, the stronger the glycemic response it can induce. In one group of pigs, we tracked how they consumed food over a period of roughly three months. This food intake behavior remained reasonably consistent throughout this period for individual pigs. Hence, we tried to correlate the number of meals, meal size and food intake to adiposity of the pigs. Figure 9A shows that an inverse relationship exists between the number of meals per day and the meal size. Multiplying these two values equals the total food intake per day. The correlation analysis (Figure 9B) indicates that food intake was positively correlated with adiposity; eating more food resulted in slightly fatter and less muscular pigs (this is very much calorie counting). The number of meals/day (i.e., the inverse of the meal size) also had an effect; pigs that ate more frequently and thus had smaller meals were less fat and more muscular. In fact, this is quite logical, given the glycemia story above. Smaller meals mean less stimulation of blood glucose and insulin; glucose ends up less in the ‘red’ (‘danger’) zone (Figure 5).

## 7. Eating Pattern

**Figure 10:**
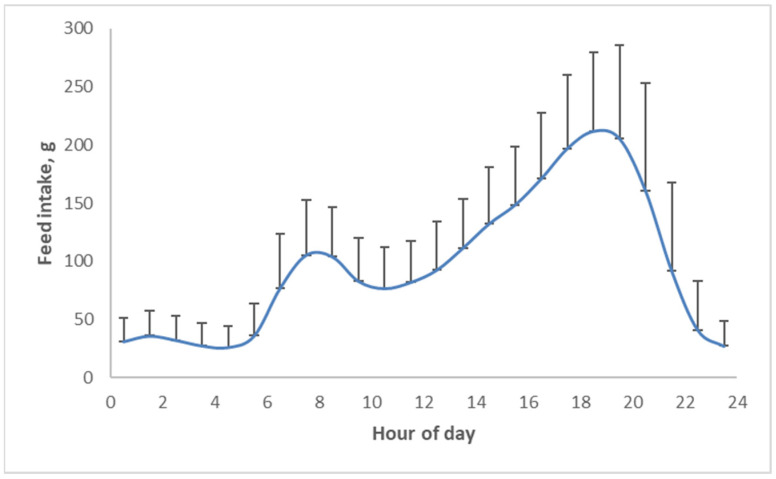
Circadian food intake pattern of pigs with continuous access to food [20].

When pigs are held in confinement with ad libitum (unrestricted) access to food, they eat numerous times throughout the day (Figure 9). These meals, though, are not randomly spread throughout the day; pigs prefer to eat ‘breakfast’, thus early in the morning, and especially dinner, thus late in the afternoon (Figure 10) [20]. This food intake pattern appears to be driven by changes in hormones throughout the course of the day, with melatonin and cortisol as the main drivers [21]. Additionally, insulin, as the key hormone in obesity, apparently induces different effects at different times during the day.

If pigs are forced to eat in a pattern, which is not natural, then the utilization of energy is changed. For example, in one experiment, we shifted the food intake pattern of piglets by 12 h. This shift caused the pigs to accrete more fat (+7%) and reduced their activity (−7%) [22]. Hence, it appears that our pigs tolerated an enormous influx of glucose late in the afternoon without it triggering adiposity. Shifting this influx of glucose to the middle of the night did trigger adiposity.

Interestingly, when group-housed pigs have limited access to food, then ‘survival of the fittest’ appears to kick in. The dominant pigs in the social hierarchy will eat food in line with the circadian profile of Figure 10. Pigs that are lower in rank, however, become much more opportunistic; they try as much as possible to eat according to the above pattern, but they seem unable to meet the desired intake during peak consumption hours and instead compensate as much as possible just after the dominant pigs stop eating for the day. The total intake of these pigs does not differ per se, but as their intake pattern is not ideally distributed throughout the day anymore, the energy consumed appears to be used more for lipogenesis, resulting in slightly more obese pigs [20]. This is in line with the ‘insurance hypothesis’ of Nettle et al. [23] on food insecurity as a driver of obesity in humans. Food insecurity leads to eating patterns, which are not optimally aligned with endocrinology, and consequently, the development of adiposity.

Fortunately, pigs are smart enough to eat when it fits best with their metabolic needs, but humans appear to have lost this ability. A big bag of potato chips late at night is timed incorrectly. Note that we are not aware of data showing what the ideal pattern of food intake should be in people. Society has such a strong influence on our behavior that ‘natural’ may well be hard to find. Primitive man arguably spent the day gathering food to prepare a meal in the late afternoon, perhaps with left-overs in the morning, so our natural pattern may have matched that of the pig reasonably well.

## 8. Hyper-Processed Food

The diet that the typical person consumes has changed drastically over time, especially in recent decades. The fraction of carbohydrates has increased, and many of these carbohydrates are categorized as hyper-processed. For example, maize contains a lot of carbohydrates in the form of starch, which is contained inside a matrix, making this starch less accessible by digestive enzymes (intact maize kernels can actually pass through a pig’s digestive tract completely intact). Maize, however, is often processed to extract this starch. The resulting starch yields a much higher glycemic index than the same amount of starch in the maize itself. Processing this starch into, e.g., glucose syrup, sometimes used in soft drinks, results in an even higher glycemic index. Generalizing, hyper-processed foods are foods that result in much stronger glycemic responses than non-processed foods. As such, they can contribute to the development of obesity, as outlined above, simply by augmenting the glycemic response.

Although we do support the opinion that hyper-processed foods can contribute to obesity, experiments with pigs do not always corroborate this. One project we executed involved processing maize into three fractions: the endosperm containing the starch, the germ containing most of the protein and the hull containing most of the fiber. We subsequently tested diets based on these three different fractions. The aim of this experiment was to design a diet that yielded less feces, as the disposal of feces can be a challenge in livestock production. Textbooks claim that diets based on only endosperm, thus hardly any fiber, cause health problems and obesity, but we did not observe this in our pigs [24,25]. In addition, this contradicts the story above on glycemic response!

Another confounding observation to the glycemia hypothesis is based on an animal feed mill in the Netherlands. This feed mill has taken circular economy to the next level. Already in Western Europe, pigs are fed diets consisting of byproducts from the food industry as their primary ingredients (e.g., coproducts obtained from wheat after extracting starch). This feed mill, though, collects all kinds of foods rejected for human consumption, and they create their swine diets using these materials. In this plant, there are sheds full of cookies, caramel, whipped cream, gummy bears, bread, etc. To turn these into a pig diet, this food material is ground and heat treated. Thus, if the cookies were hyper-processed to start out with, they are hyper-hyper-processed when fed to the pigs. Interestingly, pigs fed diets based on this food material are just as lean as pigs fed more conventional diets based on regular feedstuffs (personal communication). This would imply that hyper-processing is NOT the cause of obesity, despite what we know about the glycemic index. A high glycemic food thus seems to pose an extra challenge to humans, but by itself, it may not induce obesity.

To make matters worse, a large portion of pigs that receive these hyper-processed diets are fed with so-called liquid feeding systems. This means that the food is mixed with water into a porridge, which is distributed several times per day. In contrast to the natural feeding pattern of the pigs outlined above, when pigs are fed these liquid diets, they typically receive three or four meals per day. The timing of these meals is very much dictated by the farmer and the available food mixing and distribution equipment; little attention is given to align this with natural behavior. Worse, survival of the fittest kicks in when the pigs are fed; consequently, they generally consume this porridge in a matter of minutes. From a meal-pattern perspective, this eating pattern is not ideal because the meals are not aligned with natural endocrinology, and moreover, very large meals with a high glycemic index are consumed. Multiplying this implies that the pigs are exposed to a giant glycemic response. Nevertheless, obesity is no worse than when pigs are fed more naturally. The reason for this may be that pigs only received diets that were balanced for all nutrients.

## 9. Imbalanced Diets

**Figure 11:**
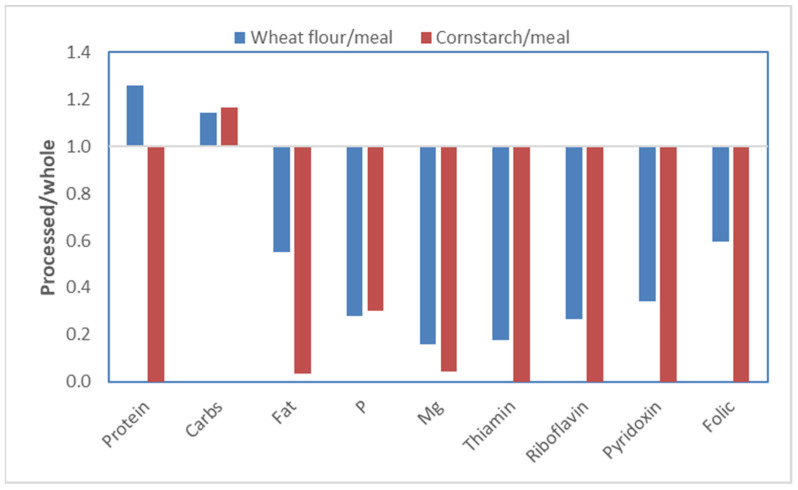
Nutrient content of wheat flour or maize starch compared to whole wheat or maize meal. Data extracted from the NEVO [27] food composition database.

The hyper-processing of food influences human food in a way that it does not affect the feed for pigs. The above experiments with the fractionation of maize can be used as an example. The germ fraction does not only contain most of the protein but also other essential nutrients, such as minerals and vitamins (referred to as micronutrients). The endosperm, in contrast, contains mostly energy in the form of starch. In Figure 11, relative nutrient and micronutrient levels are shown for wheat flour versus wheat meal (whole wheat, ground) and corn starch versus corn meal (whole corn, ground). The wheat flour or corn starch, as expected, contains more carbohydrates, mainly starch, as compared to the whole grains. The micronutrients, however, are drastically lower or even absent in the flour. It should be pointed out that, already for decades, seed companies have selected grains high in starch, which will compromise micronutrients as well [26].

When we manufactured our experimental swine diets, we took these differences in micronutrient content into consideration. Thus, we supplemented the diets based on endosperm with extra micronutrients, such that in the end, the diets were deemed identical from a micronutrient perspective! For the feed mill processing food that has been rejected for human consumption, animal diets are also formulated to be sufficient in all micronutrients!

In human nutrition, formulating individual foods for all nutrients is generally not carried out. For example, sodas, potato chips and the like are formulated for taste and product appearance. Nutritional value is not a concern. For some (primary) food products, micronutrients such as vitamins may well be added, but there is effectively no effort to balance the entire daily diet for all nutrients (meaning that the diet meets or exceeds the requirements for all nutrients known to be required for optimal growth and health). In fact, the information is not even available in the public human food composition databases, despite decades of research! Additionally, neither the USDA nor EFSA specify the requirements for all vitamins or for any of the amino acids. In line with this, diets used in human-focused obesity research may well be nutritionally inadequate. For example, Lee et al. [28] used a high-calorie atherogenic diet for Ossabaw pigs containing only 8% protein; this level of protein is unlikely to meet the amino acid requirements of the animals, which would predispose them to obesity. Detailed information on essential nutrients, however, was missing from the paper and appears missing from the website of the company that manufactured the diets (Testdiet.com; accessed on 8 January 2023). Suriano et al. [29] evaluated high-fat diets for their impact on obesity. Their high-fat diets contained 36% more calories than their control diet, but neither the micronutrients nor the protein were adjusted for this increase. In both cases, the conclusions are thus severely confounded by micronutrient/energy ratios.

In animal nutrition, we even go a step further than simply formulating a diet for nutrient content; we focus on what we call the bio-available nutrient content. This means that we painstakingly study what fraction of nutrients is absorbed from a diet, and we determine the requirement based on the absorbable amount of that nutrient. As an example, an important but often ignored micronutrient is phosphate. Phosphate is a mineral, which plays a role in effectively every metabolic pathway in our body. Above, we mentioned ATP as the energy currency. ATP stands for Adenosine Tri Phosphate, and it obtains its energy from the number of phosphate groups, which are attached to it (tri is 3). Phosphate has a strong interplay with insulin. Insulin signals to the body to increase metabolic activity as the energy is arriving from the diet, which needs to be processed somehow. This increase in metabolic activity creates a demand for phosphate. This also means that the phosphate status of a person is dependent on what the person is doing at that moment. If the person has just consumed a big bag of processed starch (e.g., potato chips), then metabolism is triggered to process this starch into heat, activity or, e.g., adipose tissue, for which phosphate is pulled from blood into cells in order to support metabolism. Processing the starch into fat apparently requires the least amount of phosphate within the body. Thus, when phosphate is in short supply, we predispose ourselves to adiposity when consuming high glycemic foods without concomitantly providing phosphate. Metabolically, this is apparently ‘the body’s easy way out.

**Figure 12:**
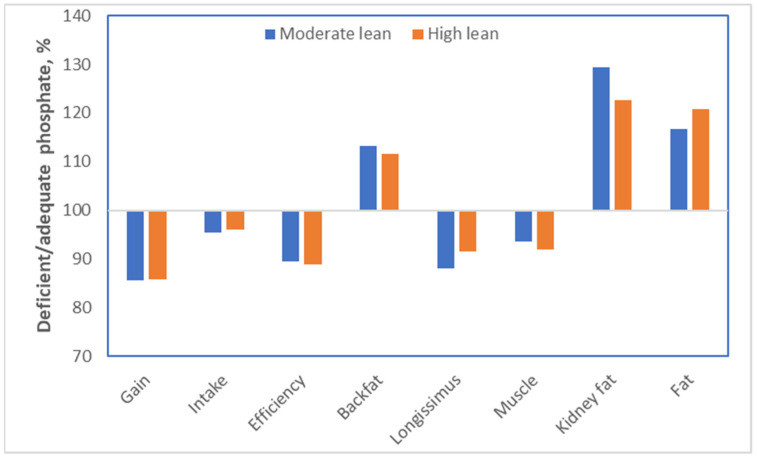
Effect of diets deficient in phosphate versus adequate amounts on performance and body composition using a moderate and a high lean genetic line of pigs [30].

For pigs, we perform experiments to determine the optimum level of each nutrient in a diet. These types of dose–response data provide interesting insights with respect to obesity. The nutrient phosphate can again be used as an example. Bertram [30] performed dose–response studies in which the bioavailable phosphate content ranged from 0.08 to 0.475% of the diet. For this experiment, two genetic lines of pigs were used: a highly muscular line and a moderately muscular line. The key results from this experiment are summarized in Figure 12. Pigs fed diets that were deficient in phosphate ate somewhat less (−4%), although not spectacularly so. Their growth, however, was strongly compromised (−14%). The conversion of food into growth, consequently, was also strongly compromised (−11%). If we go back to calorie counting, the energy intake of the pigs was thus reduced by 4%, but growth was compromised much more drastically. The explanation for this is that the energy was used to grow a tissue, which is energetically very expensive to grow: adipose tissue. Indeed, backfat increased by 12–13%, kidney fat by 23–29% (which is especially unhealthy) and total body fat by 17–21%. This fat gain occurred at the expense of muscle growth; the longissimus dorsi (or loin eye) muscle was 8–12% smaller, and the total muscle content was reduced by 6–8%. Although there were small differences between the two genetic lines of pigs, none of those were statistically significant.

Similar datasets exist for other micronutrients, both minerals and B vitamins (and for amino acids). Many of these micronutrients, when deficient, compromise metabolic health, which often hinders the body’s ability to grow muscle. The growth of adipose tissue, however, is apparently a metabolically ‘simpler’ process, and consequently, dietary energy is often funneled into adipose tissue as fat when deficiencies occur.

**Figure 13:**
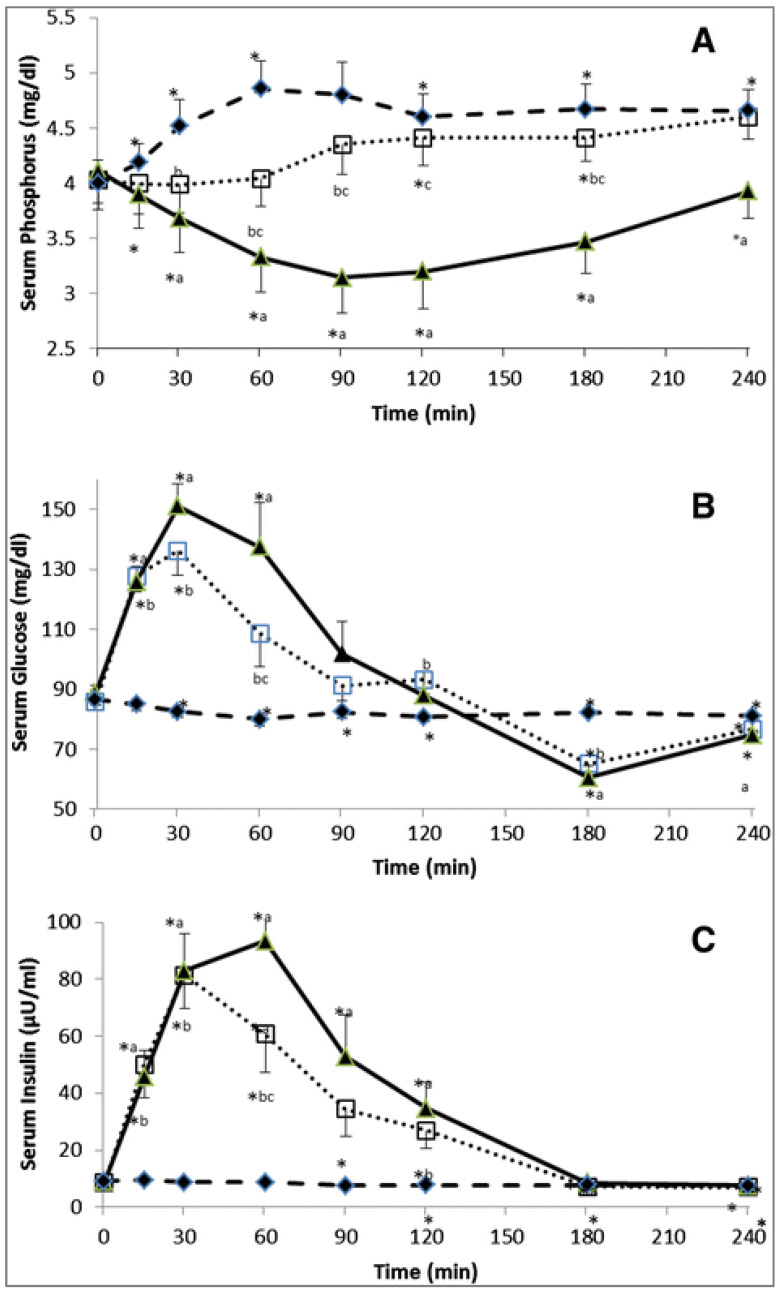
Serum phosphate (**A**), glucose (**B**) and insulin (**C**) in man in response to an oral glucose (75 g) tolerance test (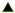), and oral phosphate (500 mg) dose (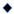), or the combination of an oral glucose tolerance test plus oral phosphate (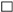). * *p*-value < 0.05: paired t-test in the same treatment in comparison with baseline (time 0) value. ^a^ *p*-value < 0.05: paired t-test, phosphorus vs. glucose treatments at each time point. ^b^ *p*-value < 0.05: paired t-test, phosphorus vs. glucose + phosphorus treatments at each time point. ^c^ *p*-value < 0.05: paired t-test, glucose vs. glucose + phosphorus treatments at each time point [31].

Consequently, based on this mechanism, the requirement for phosphate and several other micronutrients is actually a function of the glycemic load of the diet. Diets with a low glycemic load will not result in strong insulin peaks; therefore, low amounts of phosphate and related micronutrients are required to process the glucose. Diets with a strong glycemic response, however, will require a lot of phosphate and related micronutrients during this glycemic load. Khattab et al. [31] elegantly showed that blood phosphate levels were inversely related to blood glucose levels during a glycemia challenge; this drop in blood phosphate could be prevented by supplementation of phosphate at the start of the glucose challenge (Figure 13). This level of sophistication has not yet been implemented in swine nutrition, and it may well explain why diets with an extremely high glycemic index can still lead to problems with adiposity as outlined above despite formulating for all micronutrients.

**Figure 14:**
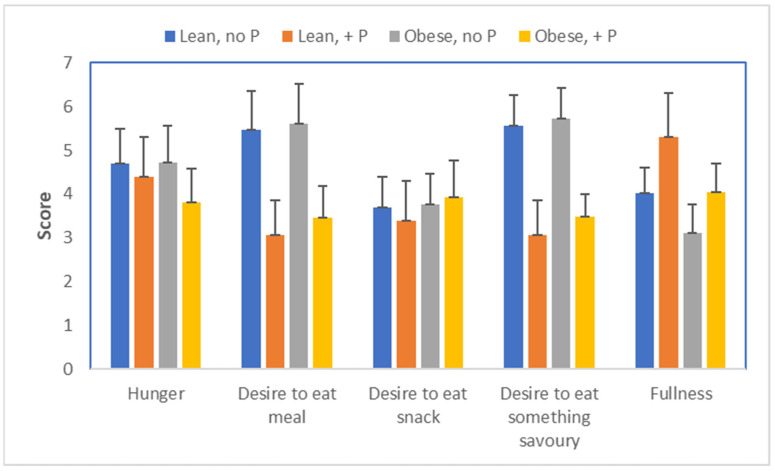
Appetite scores, 3 h after drinking a solution containing 75 g glucose, as affected by phosphate supplementation in lean and obese individuals [34].

Our bodies seem to have recognized the importance of phosphate; it is one of the few nutrients for which we have taste receptors [32]. Consequently, animals, including strict herbivores, which suffer from hypophosphatemia, will actively search for phosphate sources. These sources include bones of deceased animals (osteophagia), resulting in interesting pictures of, e.g., giraffes chewing bones. Research with lab animals confirms that induction of hypophosphatemia leads to an appetite for phosphate [33]. Bassil and Obeid [34] actually showed that appetite is also stimulated in both lean and obese humans who are hypophosphatemic (Figure 14). Their work also indicated that extra phosphate stimulated glucose oxidation in obese individuals, implying that glucose oxidation was impeded by the low phosphate status. As explained, carbohydrates that are not oxidized will likely end up being converted into fat.

**Figure 15:**
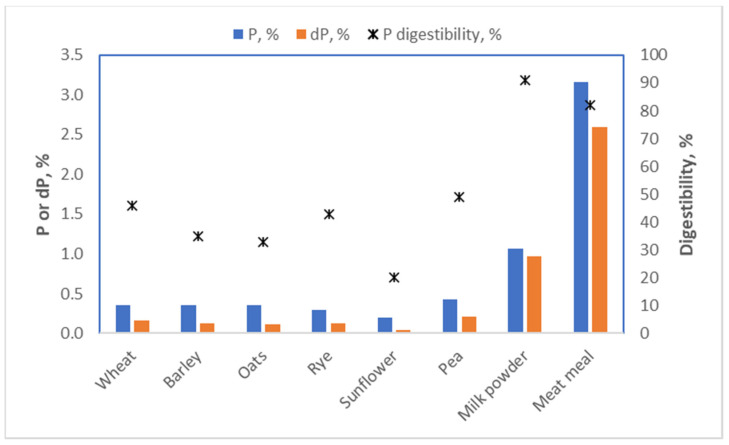
Phosphate (P), digestible phosphate (dP) content and phosphate digestibility coefficients (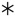) for some foodstuffs for pigs [9].

In maize and many other plants, phosphate is mainly present in the germ and stored in an ester with inositol (a sugar) called inositol phosphate or phytic acid. This nifty molecule poses a challenge for the digestive system of both humans and pigs, as neither can break it down. This means that the inositol phosphate we eat passes through the small intestine without releasing any of its phosphate, and consequently, the phosphate is not absorbed. Germ phosphate, in fact, has a digestibility in swine of only 8% [25]. Animal-derived food, in contrast, contains phosphate in a bioavailable form. Vegans are thus more prone to shortages of phosphate than omnivores. In pig nutrition, we characterized the bioavailability of phosphate for all ingredients used; therefore, we incorporated this information when formulating a diet (Figure 15). This ensures that there is no risk of our diets being deficient in phosphate. For humans, bioavailability is generally not considered, as the data are lacking. This may well mean, though, that for a diet containing ample amounts of phosphate, most of it is not absorbed, so the diet still leads to a deficiency of phosphate.

Phosphate, from a diabetes and obesity perspective, is especially interesting, as numerous reports link phosphate to insulin action. Moreover, people with type II diabetes and/or obesity are prone to be deficient in phosphate [35,36]. Although we could debate whether this is a cause or effect, the above data on hyper-processed diets fed to pigs would indicate that it is causative. After all, pigs were fed a diet, which included ‘human-targeted junk food’ but was adequate in phosphate, and they experienced no issues.

**Figure 16:**
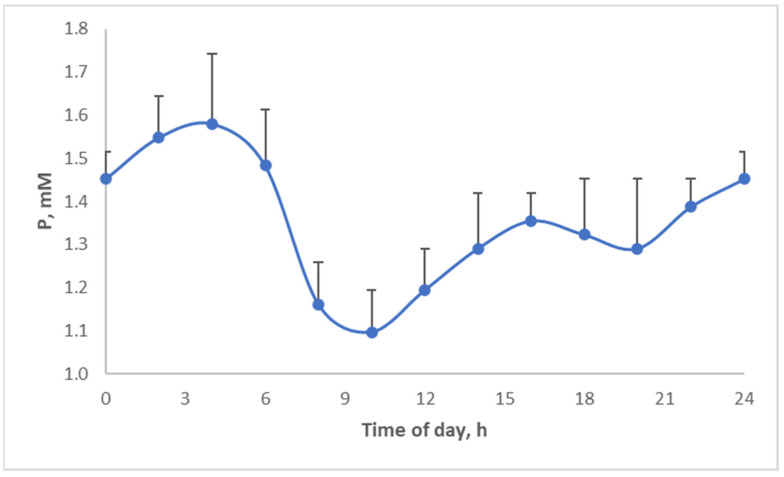
Diurnal phosphate levels [37].

Above, the diurnal food intake patterns were discussed. Several micronutrients and hormones actually display diurnal patterns. For example, phosphate shows a strong circadian pattern (Figure 16), which is actually quite similar to the food intake pattern of pigs (Figure 10) but shifted in time. Thus, by changing when we eat, we may aggravate the discrepancies between micronutrient availability and requirement.

## 10. Fructose

There are researchers who blame fructose for the obesity epidemic in mankind [38]. Johnson actually has a compelling theory that animals in the wild use a high-fructose diet to induce adiposity with the aim of building an energy buffer for winter (as well as insulation). Apples, which are high in fructose and available in the fall, fit nicely with that theory. Fructose is a sugar, which is sweeter than glucose and is present in honey, in fruit, such as apples and pears, and in some soft drinks (in copious amounts). Fructose consumption has increased strongly in recent decades, roughly in parallel with the increase in obesity, and it makes up close to 10% of our energy intake today.

Fructose is a sugar, which is hard on our bodies. Absorption from the intestines is challenging for many people, in which case, fructose can cause gut health problems. This was also shown in pigs by Ly [39], who observed that 98.3% of sucrose, 98.3% of glucose, but only 86.6% of fructose was digested in the small intestine of piglets (and the data indicated that the fructose may have predisposed piglets to diarrhea). For fructose, Ly [39] also observed a notably larger variation in digestibility between pigs. This is in line with human data, which show that some people have more problems digesting fructose than others. Fructose also taxes the liver; it is quickly turned into fructose bis-phosphate, which puts a serious strain on phosphate [40]. As reviewed by Xie et al. [41], fructose metabolism has been studied in pigs. In some but not all studies, fructose indeed compromised metabolic health and led to obesity. Thus, fructose may well be a sugar to steer away from.

## 11. Fiber

Fiber is defined as dietary components, typically carbohydrates, which are not digestible by the enzymes that humans and pigs produce themselves. Some fibers can be fermented by microbes in our digestive tract, upon which organic acids are formed, which can be absorbed and provide energy to the host. However, the microbes themselves already extract some energy. Consequently, fiber or resistant starch, per unit of weight, results in a lower ATP production in humans and pigs than, e.g., dietary glucose. Fiber therefore reduces the net energy content of a diet, and thus, the ability of the consumer to produce fat [42].

Fiber is also a material, which induces physical gut fill; it can induce the feeling of satiety. Fiber, in theory, is a great way to reduce calorie intake and prevent obesity. A downside of fiber is that, because of its structure, it can impede the digestion of other food components; and both these undigested food components and the fiber itself can trigger fermentation [43]. Although fermentation is often put in a positive light, this is not the case for everybody; in some people, fiber can induce diarrhea and flatulence.

## 12. Gut Microbiota

In recent decades, the gut microbiota has seen a surge in attention. We now know that microbes exist throughout the lumen of intestines and even within the body and that they do affect health and even wellbeing. This microbiota also interacts with carbohydrates in the diet, although especially the non-digestible carbohydrates, thus fiber. Microbiota can also play a role in obesity. Turnbaugh et al. [44] even claim that an unhealthy microbiota is responsible for obesity; transplanting these unhealthy microbes to lean subjects results in the transplant recipient becoming obese! This has resulted in numerous supplements, ranging from prebiotics that try to shift the microbiota into a healthier flora by feeding microbes, probiotics where actual beneficial microbes are fed and post-biotics where beneficial microbial metabolites are provided. These interventions are not only popular in humans to combat obesity, among other areas, but also popular in swine—although for swine, the interventions are implemented foremost to improve intestinal health.

Personally, we classify the swine-targeted products very much as ‘soft’ products; they may well have a compelling story, but it is typically hard to see the effects in the various research trials that we conducted with them. Intestinal health is an important topic, especially in newly weaned piglets, and many strategies, including manipulation of microbiota, have been tested. Generalizing, strategies that suppress the harmful microbes, such as dietary organic acids, show more consistent health benefits, including leaner animals, than strategies that try to stimulate beneficial microbes. The manipulation of microbiota in humans to combat obesity, despite several positive reports, also appears to be controversial [45].

## 13. Mediterranean Diet

Given the lower incidence of coronary heart disease in countries of southern Europe, the Mediterranean diet has received a lot of attention in human nutrition. Textbooks claim that the Mediterranean diet yields health benefits because of the consumption of olive oil and fish (important sources of oleic and omega-3 fatty acids, respectively), combined with ample vegetables, beans and whole grains. In swine nutrition, whole grains and legume seed protein are the basis for swine food in most of the world. The omega fatty acids, linoleic (C18:2 n−6) and α-linolenic (C18:3 n−3) acid, are considered nutritionally essential, just like vitamins. Consequently, their requirement during various swine life phases (e.g., infancy, pregnancy, lactation, etc.) has been determined, and formulation guidelines have been implemented. In practice, though, possibly because of the shorter lifespan of the pig, it is hard to observe deficiency symptoms, especially as they relate to obesity [9].

Interestingly, in pork production, there is also a Mediterranean diet, which may well prevent obesity. This diet, which mainly consists of acorns, is fed to the pigs that yield Iberian ham or Pata Negra. Acorns are a rich source of tannins, making them not only distasteful to people but also rendering them very hard to digest because they bind to the protein and can lead to digestive problems, i.e., diarrhea. Hence, this is not a practical solution for controlling weight in humans.

## 14. Fat: You Are What You Eat

**Figure 17:**
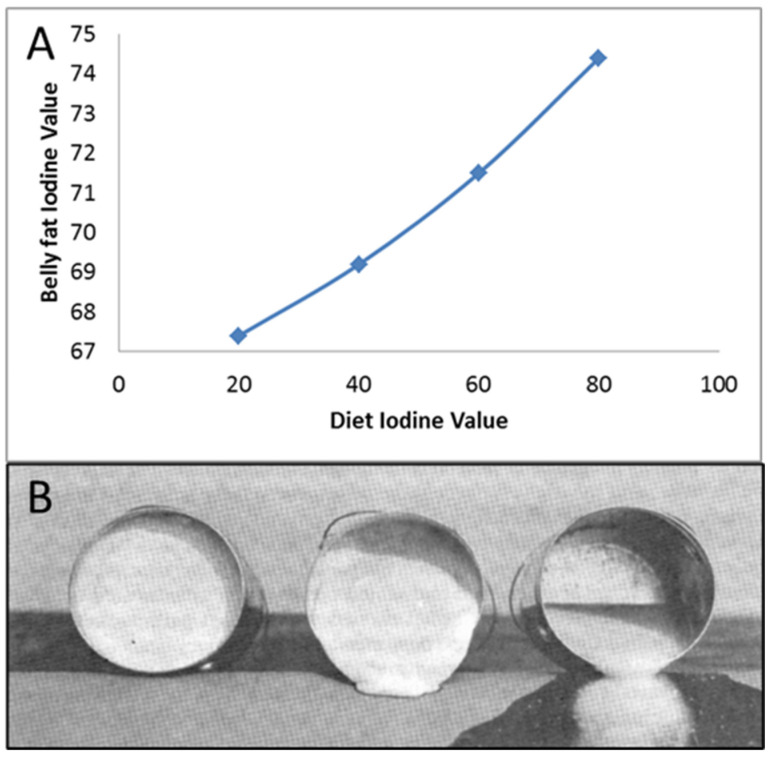
(**A**) Relationship between the fraction of unsaturated fatty acids in total fat in the diet, measured as the iodine value, and the fraction of unsaturated fat in belly fat [46]. (**B**) Pork belly fat at room temperature with low (left), medium (middle) and high iodine values (right) (image from Hankins, 1928, as used by Averette Gatlin, personal communication).

A topic, which does not receive much attention in human obesity research, but which is relevant in swine nutrition, is that ‘you are what you eat’ when it comes to fat. This statement refers to the fact that dietary fatty acids can be directly incorporated into the adipose tissue we have. Consequently, eating diets high in saturated fats results in a higher degree of saturation of adipose depots, while eating diets high in unsaturated fats results in more unsaturated fatty acids in our adipose depots. In swine, Averette Gatlin et al. [46] showed a strong relationship between the degree of fatty acid saturation in the diet and in the adipose depots of the consumer (Figure 17A). Świątkiewicz et al. [47] similarly determined correlations for the various fat classes between diet and adipose tissue and reported correlation coefficients ranging from 0.77 for mono-unsaturated fatty acids to 0.89 for poly-unsaturated fatty acids and 0.92 for iodine value as a measure of the overall degree of saturation of fats.

In pork production, this can have profound effects on meat quality. Tales circulate of swine farmers incorporating, e.g., mayonnaise, in the diets of their pigs. This mayonnaise is high in unsaturated fatty acids, so that it is liquid at room temperature. At the slaughter line, though, the fat that the pigs had accumulated was also liquid at room temperature (Figure 17B). This makes their fat very hard to slice.

Society has been pushing the consumption of unsaturated plant oils in recent decades, replacing animal fats. For example, dairy-based butter is being replaced by plant-derived margarine. As margarine contains more unsaturated fats, it is easier to spread on bread. Hence, the IV value of human adipose reserves should have also increased in recent years, resulting in fats that are more liquid at body temperature. Extrapolating from swine, in obese people, such fats are more prone to be affected by gravity and may result in, e.g., bellies, which easily hang over pants or (butt-)cheeks that droop down. Hence, they may make us look more obese.

Notably, unsaturated fatty acids are also more prone to produce free radicals during their oxidation, especially as we age. In addition, unsaturated fatty acids are susceptible to oxidative damage, in contrast to saturated fats. Hence, from an oxidative stress perspective, a key factor in coronary heart disease, unsaturated fats also pose challenges both through their ability to induce oxidative stress and due to their ability to be irreversibly damaged by oxidative stress [48].

## 15. Healthy Foods

**Figure 18:**
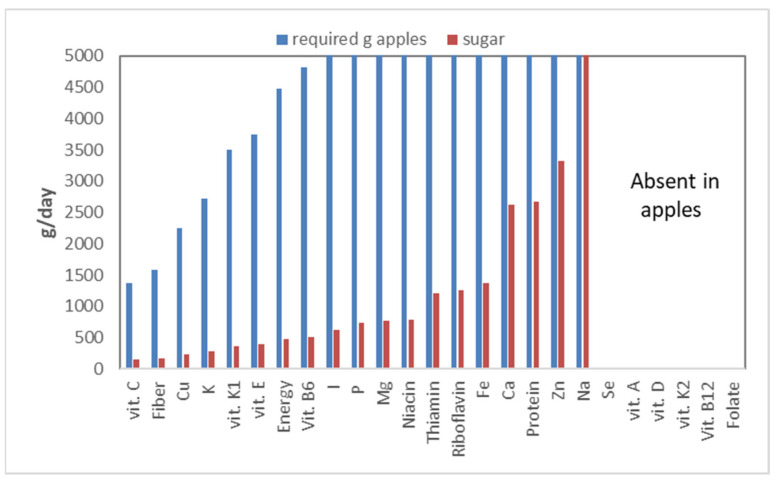
The amount of apple (g) one needs to eat to obtain enough of a nutrient to meet the daily requirement (RDI), and the amount of sugar associated with that intake. The graph is cut off at 5000 g, and from Se to Folate, the apples do not provide any value, so no daily intake can be calculated.

‘An apple a day keeps the doctor away’ is a well-known phrase in the English language. Indeed, apples are deemed as healthy by many, including many health professionals. To look at the health benefits of apples, we analyzed them in the same manner as the ingredients for use in swine diets. In order to achieve this, we took the nutritional composition, as listed in the NEVO [27] database, and the recommended dietary intake for a 40-year-old person. Based on this, we calculated for each nutrient how much apple one would have to eat each day to meet the nutritional requirement. Indeed, this is an odd calculation, as we do not live on apples alone, but it hopefully makes the numbers more tangible (Figure 18).

Apples turn out to be a reasonable source of vitamin C: one would have to eat 1375 g of apples a day to meet the vitamin C requirement. Apples are also a good source of fiber, requiring only 1575 g of apples a day. The dominant fiber in apples, pectin, is a ‘sticky’ fiber, which will not work for everybody, as it strongly stimulates fermentation. Apples, however, are also an important source of sugars: 10.5%. This number becomes even more staggering when expressed on a dry matter basis: 72%! Even worse, apples are high in fructose, a sugar that is hard on the body, as explained above. Calculating the sugar intake, which would accompany the various nutrients that apples contain, shows that to meet one’s vitamin C requirement, the total sugar intake would be 144 g, which is far above what is deemed healthy! This means that unhealthy nutrients (sugar) are relatively more important in apples than vitamin C, the nutrient for which apples are a relatively good source.

Several nutrients (selenium, folate, and vitamin A, D, K_2_, B_12_) are not provided by apples—hence no bars in Figure 18—while for iodine (I) to sodium (Na), the required intake is above 5000 g/d, or well above 500 g of sugars per day. This graph also ignores bio-availability; not all nutrients contained in apples are indeed accessible for absorption by the intestines. Thus, in reality, the required intakes should be even higher.

Apples do contain phytonutrients, such as quercetin. Quercetin is a well-researched polyphenol, which can act as a potent antioxidant, and indeed, many health claims are described for quercetin. Quercetin, though, is contained exclusively in the peel, so it would have to be eaten for any health benefits. Unfortunately, quercetin is difficult to absorb by our intestines, so an important question is whether it can provide any benefit. To our knowledge, the European regulatory agencies have not formally approved any of these health claims, and trials with quercetin in pigs failed to show any benefit. Based on this assessment, it is thus more logical to conclude that apples are very much like candy, providing some vitamin C and fiber.

## 16. Conclusions

Pigs show a great similarity to people when it comes to physiology and the type of food that they can consume. Pigs may well be fed diets, which are deemed obesogenic in humans: high in sugars or hyper-processed gelatinized starch. Moreover, the feeding patterns for pigs are also not always ideal. Nevertheless, obesity and diabetes problems in swine, managed for productive purposes, are rare.

Two key differences appear to exist between man and pig.

- Diets for pigs are optimized for all dietary nutrients, which are deemed of physiological importance. Consequently, pigs are unlikely to suffer from drastic deficiencies in, e.g., phosphate or amino acids. Such deficiencies can impair how the energy consumed is used. Converting dietary energy into fat seems to be the ‘path of least resistance’; when deficiencies in nutrients such as phosphate impede other means of energy utilization, conversion into fat seems to persist.
- Pigs seem to understand their own physiological limits much better than humans. For example, pigs have a strong preference to eat at times of the day, which align with their endocrine make-up. Additionally, pigs that have compromised insulin sensitivity due to, e.g., epigenetic reprograming seem to reduce their food intake, especially of diets with a high glycemic load, to prevent themselves from becoming obese. In humans, we seem to have lost these abilities.

Other lessons gained from working with pigs:

- Calorie counting is something we rely on strongly in swine nutrition, and there are few reasons to believe that it does not work in humans. Most humans should be able to lose weight by consuming fewer calories than what they need for maintenance and physical activity. Some variation in efficiency may well exist between people because of genotype, sex, etc., but this is likely to be small.
- A diet with a high glycemic index does not per se induce obesity, but lowering the glycemic burden of our diets should make it easier to prevent obesity. Technically, this can also be achieved by reducing the amount of carbohydrates in food, by having more frequent and smaller meals, and/or by switching to starch sources high in amylose.

Thus, should you eat like a pig to prevent obesity? Somehow, this does indeed work, despite diets that many human nutritionists would actually frown upon.

A personal view is that it is a pity that human nutritionists do not interact much with their swine colleagues with the intention to learn from each other. Both specialists are working with species, which are quite similar with respect to nutrition, digestive physiology and nutrient utilization. On the swine side, though, the knowledge on the nutrient yield of foods and the nutrient requirement of the target animal appears further advanced, and controlled feeding trials are much easier to perform (and on the human side, more fundamental studies are performed). Although a human is not a pig, borrowing pig data is arguably much closer to the truth than having no data at all.

## Figures and Tables

**Table 1:** Modeling coefficients for describing energy utilization in various genetic lines of pigs. a is the maintenance requirement; c is the fraction of metabolizable energy (ME) supplied in excess of maintenance, which is designated for protein deposition at 20 kg of body weight; d is the change in partitioning of ME toward protein deposition due to a change in body weight; k_p_ is the partial efficiency of protein deposition; and k_f_ is the partial efficiency of lipid deposition [6].

Group	a, kJ/kg^0.643^	c	d, /kg	k_p_		k_f_
Piétrain	762	0.544	0.00033	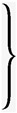	0.511	0.916
Meishan	799	0.399	−0.00302			
Large white, ♂	826	0.547	−0.00162			
Large white, ♀	868	0.561	−0.00342			
Large white, ⚦	887	0.525	−0.00368			

## Data Availability

Data sharing not applicable.
